# Aging-related fears and their associations with ideal life expectancy

**DOI:** 10.1007/s10433-021-00661-3

**Published:** 2021-11-22

**Authors:** Fiona S. Rupprecht, Kristina Martin, Frieder R. Lang

**Affiliations:** Institute of Psychogerontology, Friedrich-Alexander University of Erlangen-Nuremberg, Kobergerstr. 62, 90408 Nuremberg, Germany

**Keywords:** Ideal life expectancy, Longevity desires, Fear of death, Aging-related fears

## Abstract

Fears regarding various aspects tend to stimulate individuals to escape or to avoid the sources of the threat. We concluded that fears associated with the future aging process, like the fear of aging-related diseases, the fear of loneliness in old age, and the fear of death, would stimulate patterns of avoidance when it comes to ideal life expectancy. We expected fear of aging-related diseases and fear of loneliness in old age to be related to lower ideal life expectancies. We expected fear of death to be related to higher ideal life expectancies. In two adult lifespan samples [*N*_1_ = 1065 and *N*_2_ = 591; ages ranging from 18 to 95 years, *M* (SD)_1_ = 58.1 (17.2) years, *M* (SD)_2_ = 52.6 (18.1) years], we were able to support our hypothesis regarding fear of death. We furthermore found significant interactions among the fears, indicating that individuals fearing diseases or loneliness but being unafraid of death opted for the shortest lives. Our results indicate that fears regarding life in very old age might be associated with the wish to avoid this age period; the fear of death was however associated with the wish for particularly long lives, and thus, with distancing oneself from the dreaded event of death. We conclude that fears seem to be associated with how individuals approach old age and with what they wish for in their own future as aged people.

## Introduction

As the number of nonagenarians and centenarians is continuously rising around the world (Stepler 2016), absolute and relative numbers of retirees are growing, and diseases and disabilities are increasingly occurring in oldest age (GBD 2019 Diseases and Injuries Collaborators 2020), it becomes particularly important to understand how people envision very old age and whether they would like to reach a high age themselves. When being asked for their ideal life expectancy, the vast majority of participants in European and Northern American studies wish to experience early old age or *third age* (Ambrosi-Randić et al 2018; Bowen et al 2020; Chopik et al 2018; Lang et al 2007). This age period describes the part of an individual’s old age that is characterized by high levels of psychological, physical, and social well-being (oftentimes up to the ages of 75 or 80 years; Baltes and Smith 2003). Fewer individuals however wish to reach very old age or *fourth age* (Bowen and Skirbekk 2017; Lang et al 2007), which is the period of an individual’s old age associated with higher levels of frailty, multimorbidity, loneliness, and dependency (mostly 80 years and older; Baltes and Smith 2003). An important explanation for why some individuals limit their ideal life expectancy might thus be fears regarding living conditions in very old age. The current paper focuses on the interplay between different aging-related fears and the construct of ideal life expectancy.

Ideal life expectancy can be defined as a personal and relatively stable desire regarding the length of life (cf. Rupprecht and Lang 2020). It is assumed to influence actual survival (Karppinen et al 2012), engagement in health behaviors (Bowen et al 2020), and psychological well-being (Rupprecht and Lang 2020). As individuals form ideal life expectancies for an unknown and largely unforeseeable future, anticipations of the personal life in old age should be crucial (Awang et al 2019; Bowen and Skirbekk 2017).

Fears hereby refer to explicitly negative anticipations of a possible future. In contrast to generalized anxieties, fears relate to specific objects, situations, conditions, and states (Öhman 2008; Epstein 1972). For example, Arrindell et al (1991) identified four broad and fundamental classes of fears or phobias, that is, agoraphobic fears, fears of animals, interpersonal fears, and fears regarding death and illness (the two latter classes of fears include the aging-related fears studied within this paper). From an evolutionary perspective, fears serve the function to protect individuals from bodily, but also social and psychological harm. The most central coping mechanism to encounter fears and to protect oneself is hereby avoidance (Epstein 1972; Frijda et al 1989; Smith and Freund 2002). Individuals would thus strive to avoid the aspects of old age they fear and while a proactive way of doing so would be to engage in protective and preparatory behavior, a straightforward but passive way would be to evade the fears by wishing to not reach very old age at all. In the following, we will elaborate on this idea that specific fears regarding the aging process, that is, the fear of aging-related diseases, the fear of loneliness in old age, and the fear of death, are related to patterns of avoidance when it comes to ideal life expectancy.

### Fearing aging-related diseases and ideal life expectancy

In studies investigating fears and worries regarding a future aging process, health concerns have been at the forefront of participants’ minds (Neikrug 2003; Smith and Freund 2002; Wisocki et al. 1988). Accordingly, individuals regularly state good health as a condition for wanting to reach very old age (Brandão et al 2019; Ekerdt et al 2017; Karppinen et al 2016). When asked to trade-off between a shorter life in good health and a longer life in impaired health, many individuals seem to opt for the former option (Ayalon and King-Kallimanis 2010; Tsevat et al. 1998). Consequently, we previously identified a *medicalist* mindset for longevity motivation (Lang and Rupprecht 2019). The *medicalist* mindset is associated with an appreciation of longevity as long as life is healthy and characterized by good physical, cognitive, and psychological functioning. In relation to our research question, individuals particularly afraid of illness and aging-related diseases (e.g., dementia and stroke) might be more hesitant in wishing to reach very old age. A strong fear of aging-related diseases might thus be associated with lower ideal life expectancies.

### Fearing loneliness in old age and ideal life expectancy

Another fear commonly associated with reaching very old age is the fear of surviving close others and of being left alone. Fears of bereavement and the explicit fear of loneliness in old age were among the main reasons why individuals did not want to live up to an age of 100 years in a study by Karppinen and colleagues (2016). Indeed, the absence of a spouse, but also the presence of functional limitations seem able to explain the heightened experience of loneliness at a very high age (Luhmann and Hawkley 2016). Particularly against the backdrop of the increasing subjective importance of close social relationships at the end of life (cf. socioemotional selectivity theory; Carstensen et al 1999), the fear of loneliness in old age might thus undermine the wish to live a particularly long life. Instead, individuals fearing loneliness in old age might prefer lower ideal life expectancies, so that when they die, central roles like their work status are still relevant and close others are still alive.

### Fearing death and ideal life expectancy

As getting older is inherently associated with getting closer to the end of one’s life, the fear of death might be relevant for ideal life expectancy as well. Terror management theory proposes that humans encounter the immediate threat of death by distancing themselves from it (Pyszczynski et al 1999)—for example by denying their own vulnerability (Greenberg et al 2000), or by supporting indefinite life extension (Lifshin et al. 2018, 2019). Individuals with a strong fear of death might thus try to avoid or postpone the dreaded event by wishing for particularly long lives. Indeed, a strong fear of death was related to wanting to live longer than one personally expected to in a study by Cicirelli (2006). In recent work, we observed a similar tendency for individuals with low death acceptance (Lang and Rupprecht 2019). Accordingly, we identified an *essentialist* mindset for longevity motivation, which is characterized by the strive to conjointly overcome aging, the associated biological degeneration, and death. Whereas only a minority of individuals may actually wish to overcome death completely (Partridge et al 2011), wishing for an extraordinary but still realistic length of life might be one response to a strong fear of death.

### Concurrent fears and ideal life expectancy

The different fears described above can be present concurrently, might even be interdependent, and could jointly influence an individual’s ideal life expectancy. Previous works found that when fears of aging (including aspects of illness and loneliness) were strong, fear of death tended to be strong as well (Benton et al 2007; Bodner et al. 2015). If aging and death were feared simultaneously, the hypothesis that fear of loneliness and fear of diseases could result in a weaker desire for a long life may no longer hold true. Instead, individuals might generally be so afraid of life’s finitude (including aspects of death, disease, and loneliness) that they might wish for an overall postponed aging process and particularly long life. In contrast, when fears of aging-related diseases and loneliness in old age are strong, but fear of death is weak, the wish to die before reaching very old age might seem like an acceptable resolution. We thus explored whether fears of diseases and loneliness on the one side and fear of death on the other side interact in their association with ideal life expectancy.

### Current study

The current work explores the associations between ideal life expectancy and three different fears, that is, the fear of aging-related diseases, the fear of loneliness in old age, and the fear of death. To answer our research questions, we used two studies and samples. Study 1 focused on the fear of diseases and the fear of death in relation to ideal life expectancy. We expected a stronger fear of diseases to be related to lower ideal life expectancies, as individuals fearing aging-related diseases might wish to avoid a potentially vulnerable fourth age. In contrast, we expected a stronger fear of death to result in higher ideal life expectancies as individuals fearing death might want to distance themselves from it. Additionally, we explored the interaction of fear of diseases and fear of death in their association with ideal life expectancy.

## Study 1

### Method

#### Sample and procedure

The sample for Study 1 comes from an ongoing coronavirus-centered online study. Data collection took place in September and October of 2020, a time when coronavirus cases in Germany were slowly rising again, but there were only few measures restricting public life. Participants were recruited via email distributors of our institute, local newspaper articles, as well as postings on social media. Participants were also encouraged to promote the study within their own networks. The study received approval of compliance with ethical rules and data security law by a governmental authority of the State of Bavaria (Germany).

The sample consisted of 1085 individuals, among whom 1056 had given basic demographic information. Those 1056 participants were aged 18 to 95 years (*M* = 58.1, SD = 17.2). 70% of the participants identified as women, 29% as men, and 1% as non-binary. 65% held a university degree, 66% reported being in a stable relationship, and 26% were living alone.

#### Measures

Ideal life expectancy was assessed with the question “To what age would you like to live?” (Lang and Rupprecht 2019). As the longest human lifespan verified is 122 years, we asked participants to give realistic answers and adapted values greater than 125 years to a currently imaginable lifespan of 125 years (*N* = 8 or 0.8%; see Rupprecht and Lang 2020). We additionally excluded three answers, one due to a typing error and two due to lying below the current age of the participant.

Fear of death was assessed in a time-sensitive manner by asking to what extent the statement “When thinking of my own death, I became fearful” had applied to the participant during the last week on a scale ranging from 1 (*does not apply at all)* to 7 (*applies very much*).

Fear of aging-related diseases was assessed by asking participants whether they were afraid of dementia/cancer/stroke/heart attack/serious lung disease in relation to themselves (DAK-Gesundheit 2020; Martin et al 2021). The five diseases were chosen as all of them are aging-related, i.e., their prevalence clearly increases with age and the diseases particularly affect the quality of life among old and very old adults (e.g., Cho and Stout-Delgado 2020; GBD 2019 Diseases and Injuries Collaborators 2020; White et al 2014). Items were answered on a scale ranging from 1 (*not at all*) to 5 (*very much*). The five items formed a scale with good reliability, Cronbach’s *α* = 0.84.

Age, gender, education, life satisfaction, social satisfaction, self-rated health, and a count of medical diagnoses served as covariates to cover demographics and well-being. Several of these covariates were related to ideal life expectancy in prior research (e.g., Bowen and Skirbekk 2017; Lang et al 2007; Rupprecht and Lang 2020). Age was assessed as years since birth. Gender was coded as 0 (*female or non-binary*) or 1 (*male*). History of education and degrees were translated into ISCED categories ranging from 1 (*primary education*) to 8 (*doctoral degree*). Life satisfaction was assessed with the question “How satisfied are you with your life, all things considered?”, which participants answered on a scale ranging from 0 (*completely dissatisfied*) to 10 (*completely satisfied*) (Lucas et al 2003). Social satisfaction was operationalized as the average out of two items asking participants to rate their relationship with friends/acquaintances and family on a scale ranging from 1 (*very bad*) to 5 (*very good*) (see Böger and Huxhold 2018). The two items for social satisfaction were correlated by *r* = 0.29, *p* < 0.001. For assessing self-rated health, individuals described their own health as *bad* (1), *poor* (2), *fair* (3), *good* (4), or *very good* (5). We additionally asked for the presence of nine different diagnoses—many related to the diseases we used for the fear of aging-related diseases scale (e.g., cardiovascular disease, lung disease, cancer). The number of self-reported diagnoses served as an additional health variable and could range from 0 to 9.

#### Data analysis

Following bivariate and descriptive analyses, ideal life expectancy served as the outcome variable in a regression analysis with the covariates being entered as predictors in a first step, fear of death, and fear of aging-related diseases being entered in a second step, and their interaction in a third step. Except for gender, all predictor variables were mean-centered. R 4.0.0 (R Core Team 2020) and the packages psych, interactions, and devEMF (Johnson 2020; Long 2019; Ravelle 2019) were used for data analyses and illustration.

### Results

Table 1 depicts the descriptives and bivariate correlations of the study variables. Fear of death was rather low with a mean of 2.4 (SD = 1.8) on a scale ranging from 1 to 7. Fear of aging-related diseases was moderate with a mean of 2.8 (SD = 1.0) on a scale ranging from 1 to 5. The two fears were positively related to each other (*r* = 0.34, *p* < 0.001). They were both unrelated to ideal life expectancy in the bivariate setting. Fear of death and fear of aging-related diseases were however both related to lower life satisfaction, lower social satisfaction, worse self-rated health, and a higher number of diagnoses.^1^ Ideal life expectancies amounted to an average of 87.6 years (SD = 10.3; *Quartiles* = 82/87/90). A higher ideal life expectancy was significantly associated with older age, male gender, higher life satisfaction, and social satisfaction, as well as better self-rated health.

**Table 1:** Descriptive statistics and bivariate correlations for Study 1

Variable	M	SD	1	2	3	4	5	6	7	8	9	10
1. Fear of diseases	2.77	1.00	–									
2. Fear of death	2.42	1.84	0.34**	–								
3. Ideal life expectancy	87.64	10.25	−0.03	0.05	–							
4. Age	58.07	17.18	0.09*	0.06	0.07*	–						
5. Gender	–	–	−0.01	−0.03	0.11**	0.19**	–					
6. Education	5.56	1.82	−0.07*	−0.02	0.04	0.01	0.14**	–				
7. Life satisfaction	7.06	2.16	−0.16**	−0.17**	0.16**	0.14**	0.08*	0.07*	–			
8. Social satisfaction	3.87	0.87	−0.15**	−0.09*	0.11**	−0.06	0.02	0.11**	0.44**	–		
9. Self-rated health	3.70	0.91	−0.29**	−0.21**	0.12**	− 0.14**	−0.04	0.10**	0.39**	0.29**	–	
10. Diagnoses	1.53	1.38	0.24**	0.10*	−0.05	0.33**	0.08*	−0.08*	−0.15**	−0.16**	−0.52**	–

Self-rated health, social satisfaction, and fear of death were assessed on different scales in Study 1 and Study 2

*p < .05. **p < .001

**Fig. 1:**
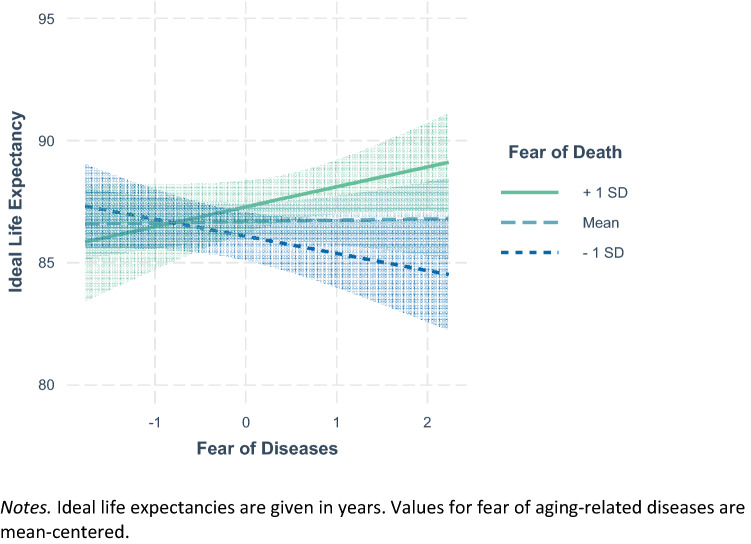
Interaction of fear of death and fear of diseases in predicting ideal life expectancy

The results of the regression analysis are depicted in Table 2. Among the covariates, a higher ideal life expectancy was related to being older, being male, reporting a higher life satisfaction, and better self-rated health. A stronger fear of death was significantly related to a higher ideal life expectancy only as long as the interaction between fear of death and fear of aging-related diseases was not part of the regression analysis. The interaction reached significance and is illustrated in Fig. 1. Ideal life expectancy was lowest when fear of aging-related diseases was strong, but fear of death was weak. Ideal life expectancy was highest when both fears were strong.

**Table 2:** Regression analysis of ideal life expectancy on fears of death and diseases

Variables	Ideal life expectancy			
	β	*B*	SE*(B)*	∆*R*^*2*^
Age	0.07*	0.04	0.02	
Gender	0.09*	2.02	0.70	
Education	0.01	0.04	0.17	
Life satisfaction	0.10*	0.47	0.17	
Social satisfaction	0.04	0.49	0.40	
Self-rated health	0.09*	1.01	0.44	
Diagnoses	−0.02	−0.16	0.28	
				4.6%**
Fear of death	0.06	0.33	0.19	
Fear of diseases	0.01	0.06	0.34	
				0.6%*
Fear of death* Fear of diseases	0.08*	0.42	0.18	
				0.5%*

Ideal life expectancy is predicted by covariates, fear of death and fear of diseases, as well as their interaction term in a stepwise procedure. Standardized regression weights β, unstandardized regression weights *B*, as well as their standard errors SE*(B)* are depicted for the final model including all predictor variables. ∆*R*^*2*^ and related F-tests are depicted for the single steps. In exploratory analyses neither self-rated health nor the count of diagnoses moderated the relationship between fear of diseases and ideal life expectancy

**p < 0.001, *p < 0.05

### Discussion of Study 1

As hypothesized, we found that a stronger fear of death was related to higher ideal life expectancies. This could reflect the wish to distance oneself from death and to push it into a more distant future. Additionally, ideal life expectancies were lowest when participants feared aging-related diseases but did not fear death. For some individuals, fears regarding certain risks of life in very old age were thus stronger than the fear of death. This combination could provoke the wish to die before reaching very old age (Bowen and Skirbekk 2017).

As these associations had not been tested in prior research and were relatively small, we seeked to validate them in a second study. Study 2 focused on fear of death and fear of loneliness in old age—another fear that targets potential risks of life in very old age (cf. Luhmann and Hawkley 2016). We again expected a positive relation between fear of death and ideal life expectancy. Following the results from Study 1, we additionally expected that ideal life expectancy would be lowest for individuals fearing loneliness in old age, but being unafraid of death. Fear of aging-related diseases and fear of loneliness in old age both target certain risks of life in very old age that individuals might be afraid of; fear of death targets death as a counterpart of life instead.

## Study 2

### Method

#### Sample and procedure

The sample for Study 2 comes from the German online study of the Aging as Future project (Rothermund et al 2012). Data included 591 adults who participated in the study in 2018 and were recruited via local and partner institutions, announcements in online platforms, as well as email distributors. The usage of the latter likely led to a small overlap of participants between Study 1 and Study 2. Participants answered questions on late-life preparation, future outlook, and subjective aging (see Kim-Knauss and Lang 2020) and were reimbursed with 20€. Participants were aged 18 to 93 years (*M* = 52.6, SD = 18.1). 58% of the participants were women, 42% were men, 60% held a university degree, 68% reported being in a stable relationship, 31% were living alone, and 15% reported a migratory background. Study 2 also received approval of compliance with ethical rules and data security law by a governmental authority of the State of Bavaria (Germany).

#### Instruments

Ideal life expectancy was assessed exactly as in Study 1. One value was excluded as it was below the current age of the participant and values exceeding 125 years of age were adapted to 125 (*N* = 8 or 1.4%). Participants were additionally asked about their perceived life expectancy with the question “To what age do you expect to live?” (Lang and Rupprecht 2019). This allowed us to investigate ideal life expectancies relative to what individuals would actually expect. The resulting construct of subjective life expectancy discordance (SLED) reflects the discrepancy between ideal and perceived life expectancy. It was calculated by subtracting ideal life expectancy from perceived life expectancy and adapting values lower than −50 to −50 (*N* = 6 or 1%; see Rupprecht and Lang 2020). Negative SLED scores indicated that participants wanted to live longer than they expected to. Positive SLED scores indicated that participants expected to live longer than they actually wanted to. SLED takes into account the expectations individuals have for the length of their life and interprets ideal life expectancies in the scope of them. For example, an ideal life expectancy of 75 years seems rather low in absolute terms; an associated SLED value of -10 years would however indicate that the ideal life expectancy is already 10 years higher than the perceived life expectancy of an individual. SLED thus offers an additional point of view on ideal life expectancies (cf. Rupprecht and Lang 2020), which should enrich the interpretation of our findings.

Fear of death was assessed with the single item “When I think of my own death, I become fearful”. Participants could indicate their agreement on a scale ranging from 1 (*I strongly disagree*) to 7 (*I strongly agree*).

Fear of loneliness was assessed with the single item “How much do you fear being lonely in old age?”. Participants answered on a scale ranging from 1 (*not at all*) to 7 (*very much*).

Age, gender, education, life satisfaction, social satisfaction, and self-rated health served as covariates. The constructs were operationalized exactly as in Study 1 with the exception of self-rated health and social satisfaction. In Study 2, individuals described their own health as *bad* (1), *poor* (2), *fair* (3), *good* (4), *very good* (5), or *excellent* (6). Social satisfaction was operationalized as the average out of the two items “How satisfied are you with your relationships with your family members?” and “How satisfied are you with your network of friends?”, which were both answered on a scale ranging from 1 (*not at all satisfied*) to 7 (*very satisfied*) (Rupprecht and Lang 2020). The two items for social satisfaction were correlated by *r* = 0.51, *p* < 0.001.

#### Data analysis

Data analyses were conducted as described for Study 1. Next to absolute ideal life expectancy, SLED served as an additional outcome variable.

### Results

Table 3 depicts the descriptives and bivariate correlations of the study variables. Fear of death and fear of loneliness were both moderate on average with means of 3.4 on scales ranging from 1 to 7. The two fears were positively related to each other, *r* = 0.27, *p* < 0.001. They were both negatively related to life satisfaction. Fear of loneliness was also related to lower social satisfaction and worse self-rated health. Both fears exhibited very small, but significant bivariate relations to ideal life expectancy. A stronger fear of loneliness in old age was related to preferring shorter life expectancies. A stronger fear of death was related to preferring longer life expectancies. Ideal life expectancies amounted to an average of 88.9 years (SD = 10.8; *Quartiles* = 85/90/95). Both fears also exhibited small, but significant bivariate relations to SLED. Greater fears of death and loneliness were both weakly related to wishing to live longer than one expected. The mean of SLED was −5.5 (SD = 9.7; *Quartiles* = −10/− 3.5/0), indicating that average participants wished to live 5.5 years longer than they expected to.

**Table 3:** Descriptive statistics and bivariate correlations for Study 2

Variable	*M*	SD	1	2	3	4	5	6	7	8	9	10
1. Fear of loneliness	3.44	1.81	−									
2. Fear of death	3.36	1.80	0.27**	–								
3. Ideal life expectancy	88.86	10.83	−0.10*	0.10*	–							
4. SLED	−5.45	9.73	−0.08*	−0.20**	−0.61**	–						
5. Age	52.63	18.08	− 0.17**	−0.13*	0.04	0.24**	–					
6. Gender	–	–	−0.11*	−0.09*	0.07	−0.06	−0.01	–				
7. Education	5.42	1.93	0.03	−0.01	0.01	0.03	−0.04	0.02	–			
8. Life satisfaction	7.25	2.01	−0.29**	−0.12*	0.14**	0.18**	0.27**	0.03	0.07	–		
9. Social satisfaction	5.31	1.32	−0.28**	−0.07	0.11*	0.10*	0.16**	−0.01	0.09*	0.49**	–	
10. Self-rated health	3.06	1.10	−0.11*	−0.06	0.14**	0.06	−0.27**	0.01	0.12*	0.30**	0.12*	–

SLED = subjective life expectancy discordance. Self-rated health, social satisfaction, and fear of death were assessed on different scales in Study 1 and Study 2

*p < 0.05, **p < 0.001

**Fig. 2:**
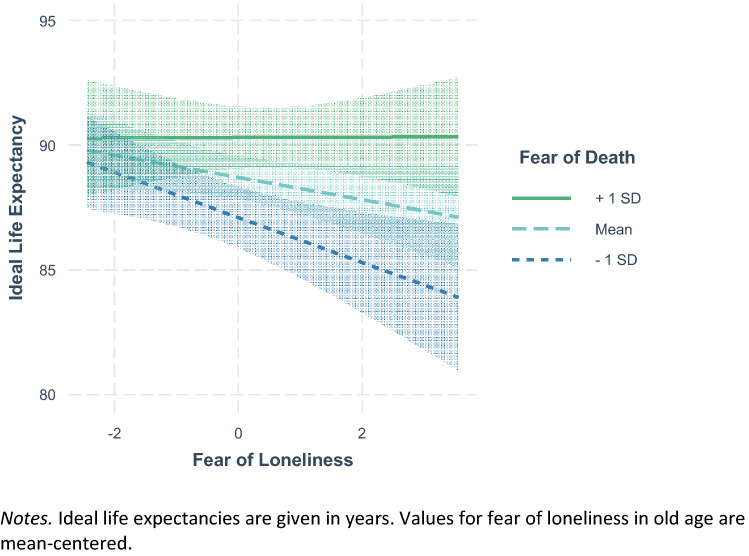
Interaction of fear of death and fear of loneliness in predicting ideal life expectancy

**Fig. 3:**
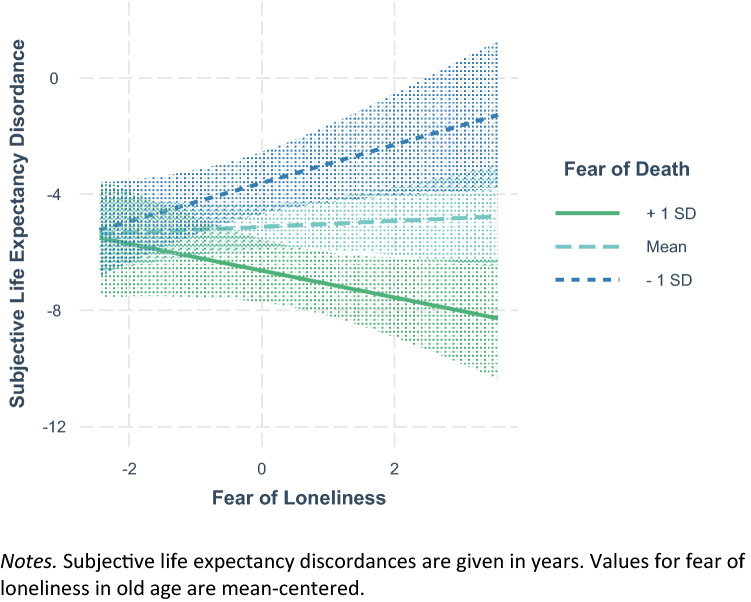
Interaction of fear of death and fear of loneliness in predicting subjective life expectancy discordance

The results of the two regression analyses are depicted in Table 4. A higher ideal life expectancy was significantly related to being male, being in better health, and being more fearful of death. Fear of death and fear of loneliness interacted in their association with ideal life expectancy. Figure 2 shows that ideal life expectancy was lowest when fear of loneliness in old age was strong, but fear of death was weak. A more negative SLED—indicating that individuals would like to live longer than they expected to—was related to a younger age, being male, and a stronger fear of death. Figure 3 depicts the significant interaction of fear of death and fear of loneliness in their association with SLED: Reporting a weak fear of death but a strong fear of loneliness was related with wanting to live approximately as long as one expected to. Moderate fear of death was related to wanting to live about five years longer than one expected, irrespective of fear of loneliness. A strong fear of death was related to wanting to live longer than one expected, particularly when there was a strong fear of loneliness as well.

**Table 4:** Regression analyses of ideal life expectancy and subjective life expectancy discordance on fears of death and loneliness

Variables	Ideal life expectancy				Subjective life expectancy discordance			
	*β*	*B*	SE(*B*)	∆*R*^2^	*β*	*B*	SE(*B*)	∆*R*^2^
Age	0.06	0.03	0.03		0.22**	0.12	0.02	
Gender	0.09*	1.98	0.87		−0.09*	−1.64	0.76	
Education	−0.01	−0.08	0.22		0.03	0.16	0.20	
Life satisfaction	0.06	0.29	0.27		0.07	0.34	0.24	
Social satisfaction	0.05	0.43	0.38		0.01	0.07	0.32	
Self-rated health	0.13*	1.30	0.44		0.09	0.74	0.39	
				4.3%**				8.8%**
Fear of death	0.15**	0.89	0.25		−0.16**	−0.83	0.22	
Fear of loneliness	−0.08	−0.45	0.26		0.02	0.10	0.23	
				2.3%**				2.6%**
Fear of death* Fear of loneliness	0.08*	0.25	0.13		−0.11*	−0.31	0.11	
				0.7%*				1.2%*

Ideal life expectancy and subjective life expectancy discordance are predicted by covariates, fear of death, and fear of loneliness, as well as their interaction term in a stepwise procedure. Standardized regression weights *β*, unstandardized regression weights *B*, as well as their standard errors SE(*B*) are depicted for the final model including all predictor variables. ∆*R*^2^ and related F-tests are depicted for the single steps

**p < 0.001, *p < 0.05

## General discussion

In both samples, average ideal life expectancies were in the late eighties and interindividual variances were of considerable size. The distributions of ideal life expectancy (lower quartiles at 82 and 85 years of age) indicate that the question whether an individual would or would not like to experience very old age indeed seems to be a relevant one. Consistently across studies, men, participants in better self-rated health, and participants who feared death more strongly wished for longer lives. Study 1 found no direct relation between fear of aging-related diseases and ideal life expectancy. In Study 2, fear of loneliness in old age was related to lower ideal life expectancies only in the bivariate setting. However, interactions between those two fears and the fear of death were significant and indicated that when risks associated with life in very old age were feared but death was not, ideal life expectancies were particularly low. In the following, we will discuss the role and implications of different fears for ideal life expectancy.

A stronger fear of death was related to a higher ideal life expectancy and wishing to live longer than one expected in our analyses. In Study 1, the relation between fear of death and ideal life expectancy was weaker and only marginally significant as soon as the interaction with fear of diseases was entered into the regression. The globally worded item for fear of death used in Study 2 was more consistently related to ideal life expectancy than the time-sensitive item used in Study 1. Nevertheless, there was a tendency in both studies to wish for longer lives when fear of death was high, supporting our hypothesis that wishing for a long(er) life might be one way of avoiding and distancing oneself from the dreaded event of death. Indeed, pushing death into a distant future and denying one’s vulnerability is thought of as a proximal defense mechanism against the threat of death (Pyszczynski et al 1999). Wishing for a long life might thus not be solely based on an appreciation of life in very old age, but also on the simple wish not to die any time soon. In contrast, being relatively unafraid of death might make individuals more accepting of scientific and personal expectations regarding longevity.

The relational patterns of fear of loneliness in old age and fear of aging-related diseases were more complex. First, both fears were significantly and positively related to fear of death, indicating that individuals fearing certain risks of life in very old age, were also more afraid of death. This finding is in accordance with previous research on interrelations between aging anxieties and death anxieties (Benton et al 2007; Bodner et al. 2015). The two fears had no direct relations with ideal life expectancy in the multivariate setting. Instead, their interactions with fear of death reached significance. In all our analyses, ideal life expectancies were lowest when fears regarding potential risks of life in very old age (i.e., loneliness and aging-related diseases) were strong, but fear of death was weak. Hence, individuals fearing what very old age might bring but being unafraid of death were the ones who expressed wishes of dying rather early. This hints at the mindset Lang and Rupprecht (2019) labeled as *medicalist*, that is the valuation of a healthy life over a long life. It also shows that fears regarding the aging process do not automatically result in lower ideal life expectancies, but do so when an earlier death seems like an acceptable resolution.

Another interesting finding pertains to those individuals who had strong fears of both death and risks associated with life in very old age. Those participants were namely the ones wishing for the longest lives and to live clearly longer than they expected. We consider this an indication of *essentialist* mindsets (Lang and Rupprecht 2019; see also Weiss et al 2016), as individuals disregarding both aging and death should be the ones with the strongest desire to overcome them completely and live particularly long or even indefinitely. As already found in previous work (Bowen et al 2020; Lang and Rupprecht 2019), men were more likely to present high ideal life expectancies than women. Reasons for this could lie in women’s stronger religiosity, afterlife beliefs, and acceptance of life’s finitude (Lifshin et al 2019), but also in differing societal roles and expectations for men and women in (very) old age (e.g., women as givers rather than recipients of care; Arber et al 2008).

### Implications and future research

The present research indicates that different fears can be associated with patterns of avoidance when it comes to ideal life expectancy—either wanting to avoid risks of life in very old age or wanting to avoid death. Importantly, particularly strong fears regarding the (future) aging process might even result in death wishes and ideation among older adults (van Wijngaarden et al 2019). One implication that arises is that interventions targeting such fears might help individuals to approach (very) old age in a more positive way and to choose a motivating ideal life expectancy. For this, personal fears could be directly addressed. Although loneliness and the prevalence of aging-related diseases do indeed increase in very old age, they are not inevitable parts of the aging process (Dykstra 2009; White et al 2014). Communicating the aging process as malleable might help to alleviate personal aging-related fears. Additionally, individuals could be encouraged and supported to cope with their fears in more active ways, for example by investing in preparatory behavior (Bowen et al 2019).

In our study, we focused on the implications of fears for ideal life expectancy. Clearly, hopes regarding the aging process could also be of relevance for ideal life expectancies. Adams-Price et al. (2016) identified two distinct, but related subscales for hopes and fears regarding personal longevity. Studies on hoped-for and feared-for future selves have argued that the balance between hopes and fears might be particularly relevant for motivation and goal-setting (Markus and Ruvolo 1989). Future research could thus jointly target hopes and fears regarding the aging process and their associations with ideal life expectancy.

Eventually, another important avenue for future research considers the perception of others who have already lived past the age one personally considers ideal. Would those others be seen as fortunate or unfortunate? Would interactions with individuals of high ages be affected? And how do personal aging-related fears relate to the ways in which older adults are approached?

### Limitations

Our work comes with a number of limitations and related opportunities for future research. First off, the effect sizes found were small, particularly in Study 1. One reason for this should be the large interindividual variances in ideal life expectancy. Also, data collection for Study 1 took part during a pandemic, which could mean that fears and their relations to ideal life expectancy were somewhat distorted. Additionally, both samples appear to reflect heterogeneity with regard to household size and migratory background but oversampled women, university-educated individuals, and homeowners (cf. Statistisches Bundesamt 2021). Results may thus not be transferable to individuals living under less privileged conditions. Data analyses were cross-sectional, which limits our findings in two regards. First, the causality behind the relationships we found could not be determined. Second, fears regarding the future (aging process) are variable over time (Smith and Freund 2002) and future research could explore how ideal life expectancies change in accordance with aging-related fears.

### Conclusion

In both studies, we observed average ideal life expectancies in the late eighties with crucial interindividual differences. Next to gender and self-rated health, the interplay of fears regarding diseases or loneliness on the one side and fear of death on the other side was able to partly explain why some individuals prefer shorter lives and others prefer longer lives. Individuals with a strong fear of death wished to live the longest lives, whereas individuals fearing aging-related diseases or loneliness in old age but being unafraid of death wished to live the shortest lives. Helping individuals to actively cope and address their respective aging-related fears might thus allow them to embrace life in old age and wish for a realistic but also motivating length of life.

## Footnotes

1 When investigating the specific subgroups of childless individuals, individuals with migratory background, and individuals living alone, fear of aging-related diseases was significantly elevated among individuals living alone. Fear of loneliness in old age was significantly elevated among childless individuals and individuals with migratory background. There were no consistent patterns for fear of death.
